# The influence of low signal-to-noise ratio of axial length measurement on prediction of target refraction, achieved using IOLMaster

**DOI:** 10.1371/journal.pone.0217584

**Published:** 2019-06-06

**Authors:** So Jung Ryu, Du Roo Kim, In Seok Song, Yong Un Shin, Mincheol Seong, Heeyoon Cho, Min Ho Kang

**Affiliations:** 1 Department of Ophthalmology, Hanyang University College of Medicine, Seoul, South Korea; 2 Department of Ophthalmology, Hanyang University Guri Hospital, Guri, South Korea; 3 Seoul Eye Clinic, Goyang, South Korea; 4 Wilmer Eye Institute, Johns Hopkins Medical Institutions, Baltimore, Maryland, United States of America; National Taiwan University Hospital, TAIWAN

## Abstract

**Purpose:**

To evaluate the influence of low signal-to-noise ratio (SNR) of axial length measurement, achieved using IOLMaster, on prediction of target refraction.

**Methods:**

A total of 131 eyes of 131 patients who underwent phacoemulsification with posterior chamber lens implantation were enrolled. Preoperative axial length measurements were performed with the IOLMaster 500 (Carl Zeiss Meditec, Germany); preoperative SNR values were used to divide the eyes into three groups (Group 1; SNR <10, Group 2; 10 ≤ SNR <50, Group 3; 50 ≤ SNR <100). One month and 6 months after cataract surgery, the manifest refraction spherical equivalents (MRSE) were measured. The mean numeric errors (MNE), the mean of the difference between postoperative MRSE, and preoperative target refraction, using the various intraocular lens (IOL) formulas, were calculated and compared among the three groups.

**Results:**

One month after cataract surgery, postoperative MRSE was more hyperopic than preoperative target refraction, calculated by the Haigis formula in group 1, and by the SRK/T formula in group 2. After 6 months, for all formulas in group 1, there were significantly hyperopic results (approximately 0.35 diopter). Upon comparison of MNE among the three groups, group 1 was statistically significantly different from the other groups by Haigis formula.

**Conclusions:**

When the SNR values in biometry, using IOLMaster, are <10, careful attention should be given to determining IOL power, as postoperative spherical equivalents are more hyperopic than preoperative target refraction by IOL formula.

## Introduction

The precise measurement of the axial length (AL) and corneal power is important in the calculation of intraocular lens (IOL) power.[1, 2] As commonly known, an inaccurate AL measurement results in crucial errors in postoperative refraction; thus, more accurate measurement of AL has been required to respond the increasing demand for cataract surgery as a refractive correction.[3] Until the 1990s, measurements of AL were generally performed by A-scan ultrasound, which has a low repeatability and can change by pressing the cornea or alternating observers.[4–6] After development of laser interferometry, such as IOLMaster, it has been widely used to decide the IOL power.[7] IOLMaster has been a useful, uncomplicated, non-contact device for calculation of the required IOL power and optimization of the A-constant by measurement of the AL, radius of corneal curvature, and anterior chamber depth.[5, 6, 8–15]

Signal-to-noise ratio (SNR) is an indicator of the accuracy of AL measurement by the IOLMaster. A high SNR value reflects higher accuracy and could help inexperienced operators choose IOL power easily.[16] In the early usage of IOLMaster, the averaging of consecutive scans was used to increase SNR, but had a limitation in that actual signals were low in amplitude. After development of version 5, the composite method (achieved by digital processing of signals of multiple measurements) had improved SNR.[17–19]

However, even with the precise measurement of IOLMaster, in cases of media opacity such as dense or severe posterior subcapsular cataract, vitreous hemorrhage, corneal opacity, and/or a patient’s poor fixation, failures to achieve high SNR occurred.[20–22] To our knowledge, there is no published study regarding how accurate the measurements of IOLMaster are, in such cases of low SNR. The purpose of our study was to evaluate the influence of low SNR on AL measurement using IOLMaster to predict target refraction.

## Methods

### Participants

This retrospective study was approved by the Institutional Review Board of Hanyang University Guri Hospital, Gyunggi-do, Korea. Ethical clearance was obtained from the Institutional Review Board (IRB no. 2017-10-019) of Hanyang University. We fully anonymized all patient information to be unidentifiable codes. All research conducted adhered to the tenets of the Declaration of Helsinki. 131 eyes of 131 consecutive patients were included from 30 to 90 years old. The patients underwent uncomplicated cataract surgery between November 2014 and April 2017 in Hanyang University Guri Hospital. Eyes with other ocular diseases, such as glaucoma, age-related macular degeneration, retinal vascular occlusion, and epiretinal membrane, were excluded. We included eyes with IOLMaster-measured SNR < 100 and those with no measurement by IOLMaster, AL > 21 mm and < 25.5 mm, and keratometry (K) values between 41 and 47 D.

### Preoperative and postoperative evaluation

All patients underwent a complete ophthalmic evaluation before surgery, including logMAR best corrected visual acuity (BCVA), severity of cataract using the Lens Opacities Classification System (LOCS III)[23], preoperative manifest refraction spherical equivalent (MRSE), and AL and K values using IOLMaster. The same examiner (M.H.K) estimated the dilated lens condition, based on LOCS III before surgery, using the slit-lamp. The examiner also measured MRSE at 1 and 6 months after cataract surgery, as well as mean numeric error (MNE; mean of the difference between postoperative MRSE and preoperative target refraction).

### IOLMaster measurement

We used IOLMaster 500 (Carl Zeiss Meditec, Germany), performed by two certified ophthalmic technicians. They performed IOLMaster for each patient 5–20 times, in order to obtain the best measurement, as defined by the highest SNR. There was no manual selection of the peak in low SNR measurements. Regular settings for phakic eyes were used and all measurements were obtained in undilated eyes. The Haigis, SRK/T, and Hoffer Q formulas were used to calculate the IOL power, using IOL constants in the User group for Laser Interference Biometry. The target refraction was selected in consultation between Surgeon(M.H.K) and his assistant resident within emmetropia to -0.50 D.

### Surgical technique

All cataract surgeries were performed by the same experienced surgeon (M.H.K.), using phacoemulsification with posterior chamber intraocular lens implantation. Preoperative pupil dilation was performed with a combination of topical tropicamide 0.5% and phenylephrine 0.5% (Tropherine, Hanmi Pharm.) The anesthesia was performed using topical proparacaine hydrochloride 0.5% (Alcaine, Alcon) and 4% lidocaine. After 2.8-mm incision was made, intracameral 1% lidocaine was injected, followed by injection of 1% sodium hyaluronate (Healon, AMO, Los Angeles, California, USA) or 3% sodium hyaluronate plus 4% chondroitin sulfate (Viscoat, Alcon, Puurs, Belgium). The Tecnis ZCB00 1-piece IOL (Abbott Medical Optics Inc.), EnVista MX60 1-piece IOL (Bausch and Lomb Incorporated) and AcrySof IQ SN60WF 1-piece IOL (Alcon Inc.) were implanted following creation of a continuous curvilinear capsulorhexis and phacoemulsification.

### Statistical analysis

We divided the patients into three groups for analysis, according to SNR as follows: SNR < 10 (group 1), 10 ≤ SNR <50 (group 2), 50 ≤ SNR <100 (group 3). All statistical analyses were performed using SPSS version 22.0 (SPSS Inc., Chicago, IL, USA)). The Kruskal-Wallis U test was used to reveal the association between BCVA and SNR. The Wilcoxon signed-rank test was used to analyze MNE at 1 and 6 months after cataract surgery. For comparing MNE between each group, Shapiro-Wilk test was used to confirm normal distribution, and one-way ANOVA was applied. Statistical significance was defined as *P* < 0.05. To reduce the bias from the variety of IOLs, one-way ANOVA was used to compare the MNE in the groups with three different types of IOLs.

## Results

### Demographic data

A total of 47 eyes were excluded because of other ocular pathologies (25 eyes), or because of high or low axial lengths (14 eyes) and K values (8 eyes). Ultimately, 131 eyes were included in the analysis. There were no significant differences in age, sex, laterality, except in preoperative BCVA among the three groups. Significantly lower BCVA was observed in group 1 (*p* = 0.039) (Table 1). The Tecnis ZCB00, EnVista MX60, and AcrySof IQ SN60WF were inserted in 72 eyes, 42 eyes, and 17 eyes, respectively.

**Table 1:** Demographics and ocular characteristics of participants.

	Total	Group 1 (SNR < 10)	Group 2 (10 2SNR <50)	Group 3 (50 3SNR <100)	*P* value
**Number of eyes**	131	30	52	49	
**Age, y**	70.9 y of e	72.2 y of e	70.2 y of e	70.0 y of e	*0*.*533*
**Sex, male:female**	63:68	16:14	25:27	22:27	*0*.*388*
**Laterality, OD:OS**	64:67	13:17	30:22	21:28	*0*.*424*
**BCVA, logMAR**		1.01 RlogMA	0.48 RlogMA	0.28 RlogMA	*0*.*039*

SNR = Signal-to-Noise Ratio; BCVA = best-corrected visual acuity. P value by Kruskal-Wallis u test: between 3 groups

### The signal-to-noise ratio

The mean preoperative SNR was 48.87 ± 16.42 in all patients. The mean preoperative SNR of group 1 was 6.61 ± 2.33 (n = 30), while those of groups 2 and 3 were 31.15 ± 10.87 (n = 52), and 75.31 ± 14.54 (n = 49).

### MNE: 1 month and 6 months after cataract surgery

Table 2 shows MNE 1 and 6 months after cataract surgery in the three groups, using the Haigis, SRK/T, and Hoffer Q formulas. At 1 month, postoperative MRSE was more hyperopic than preoperative target refraction, calculated by Haigis formula, in group 1 (+0.16 ± 0.33, *p* = 0.047). At 6 months, for all formulas in group 1, there were significantly hyperopic results (Haigis; +0.37 ± 0.31, *p* = 0.010, SRK/T; +0.31 ± 0.35, *p* = 0.017, Hoffer Q; +0.38 ± 0.49, *p* = 0.013). At 6 months, the MNE of group 1 was approximately 0.35 D hyperopic in all formulas.

**Table 2:** The mean numeric errors using 3 kinds of formula after 1 month and 6 months.

		1 Month		6 Month	
		MNE	*P* value	MNE	*P* value
Group 1	Haigis	+0.16 1 mean	*0*.*047*	+0.37 1 mean	*0*.*010*
	SRK/T	+0.07 1 mean	*0*.*487*	+0.31 1 mean	*0*.*017*
	Hoffer Q	+0.09 Qmean	*0*.*282*	+0.38 Qmean	*0*.*013*
Group 2	Haigis	-0.17 2Qmean	*0*.*175*	+0.07 2Qmean	*0*.*798*
	SRK/T	+0.12 2Qmean	*0*.*051*	-0.04 2Qmean	*0*.*502*
	Hoffer Q	+0.02 Qmean	*0*.*588*	+0.06 Qmean	*0*.*467*
Group 3	Haigis	-0.16 3Qmean	*0*.*160*	-0.10 3Qmean	*0*.*261*
	SRK/T	-0.02 3Qmean	*0*.*119*	-0.10 3Qmean	*0*.*176*
	Hoffer Q	-0.10 Qmean	*0*.*098*	-0.03 Qmean	*0*.*267*

MNE = mean numeric errors, P value by Wilcoxon signed-rank test

### MNE: Comparison among three groups according to SNR

In comparison of MNEs among the three groups, group 1 was statistically significantly different from the other groups when the Haigis formula was applied after 1 month (+0.32 ± 0.14 [*p* = 0.048] compared with group 2, +0.32 ± 0.15 [*p* = 0.047] compared with group 3). At 6 months, group 1 also showed significant differences according to the Haigis, SRK/T, and Hoffer Q formulas, compared with group 3 (Haigis; +0.48 ± 0.16, *p* = 0.017, SRK/T; +0.44 ± 0.12, *p* = 0.029, Hofer Q; +0.40 ± 0.20, *p* = 0.046). Groups 2 and 3 did not reveal statistical differences according to any of the formulas at 1 and 6 months (Table 3). Table 4 shows that there was no statistical difference among the groups with three different IOLs.

**Table 3:** The comparision of mean numeric errors between 3 groups.

		1 Month		6 Month	
		MNE	*P* value	MNE	*P* value
Group 1 vs 2	Haigis	+0.32 1 vs 2	*0*.*048*	+0.30 1 vs 2	*0*.*167*
	SRK/T	-0.05 1 vs 2	*0*.*505*	+0.35 1 vs 2	*0*.*172*
	Hoffer Q	+0.08 Qvs 2	*0*.*577*	+0.29 Qvs 2	*0*.*296*
Group 1 vs 3	Haigis	+0.32 1 vs 3	*0*.*047*	+0.48 1 vs 3	*0*.*017*
	SRK/T	+0.16 1 vs 3	*0*.*378*	+0.44 1 vs 3	*0*.*029*
	Hoffer Q	+0.20 Qvs 3	*0*.*197*	+0.40 Qvs 3	*0*.*046*
Group 2 vs 3	Haigis	-0.01 2 vs 3	*0*.*978*	+0.11 2 vs 3	*0*.*804*
	SRK/T	+0.13 2 vs 3	*0*.*797*	+0.05 2 vs 3	*0*.*886*
	Hoffer Q	+0.12 Qvs 3	*0*.*673*	+0.06 Qvs 3	*0*.*894*

MNE = mean numeric errors, P value by one way ANOVA. Here, MNE refers to the former group–the latter group

**Table 4:** The comparison of mean numeric errors between 3 kinds IOLs.

		1 Month		6 Month	
		MNE	*P* value	MNE	*P* value
ZCB00 vs EnVista MX60	Haigis	-0.12 a MX60	*0*.*460*	-0.08 a MX60	*0*.*482*
	SRK/T	+0.04 a MX60	*0*.*799*	+0.06 a MX60	*0*.*506*
	Hoffer Q	-0.07 QMX60	*0*.*687*	-0.10 QMX60	*0*.*246*
ZCB00 vs AcrySof IQ SN60WF	Haigis	+0.01 60WF S	*0*.*976*	-0.04 60WF S	*0*.*676*
	SRK/T	-0.02 60WF S	*0*.*961*	-0.02 60WF S	*0*.*906*
	Hoffer Q	-0.11 QWF S	*0*.*510*	-0.09 Q0.47	*0*.*576*
EnVista MX60 vs AcrySof IQ SN60WF	Haigis	+0.06 60WF S	*0*.*699*	-0.01 60WF S	*0*.*974*
	SRK/T	+0.01 60WF S	*0*.*989*	+0.05 60WF S	*0*.*704*
	Hoffer Q	-0.11 QWF S	*0*.*398*	-0.10 QWF S	*0*.*401*

MNE = mean numeric errors, P value by one way ANOVA. Here, MNE refers to the former group–the latter group

## Discussion

Our study showed postoperative hyperopia in group 1 according to the Haigis formula and in group 2 by the SRK/T formula, at 1 month postoperatively. At 6 months, in group 1, the postoperative MRSE was approximately 0.35 D more hyperopic than the preoperative target refraction, as calculated by all formulas. Group 1 exhibited statistically significant differences using the Haigis formula, compared with groups 2 and 3. We also found a correlation of worse preoperative visual acuity with low SNR.

As mentioned above, a significant problem in laser optical biometry is the failure of measurements. Before enrollment in this study, 11.0% of eyes were excluded because of the failure of measurement. This is a similar proportion as reported in previous studies.[10, 14, 16, 24] Further, the insufficient achievement of SNR values is a disadvantage of IOLMaster. After developing a composite scan, the proportion of eyes with insufficient SNR was markedly reduced by the averaging of consecutive optical scans.[17] In spite of the composite algorithm, eyes with low SNR remained; the selection of their IOL power is a difficult problem for many cataract surgeons. In cases of low SNR, the accompanying A-scan mode of ultrasonography (US) is helpful. However, the low repeatability and reproducibility are major limitations of this method; IOLMaster, despite the low SNR, is considered as a first-line instrument for AL measurement.

The quality and reliability of IOLMaster measurement were associated with various factors, and the SNR indicates the accuracy of the measurements.[16] We found that low SNR was associated with worse preoperative BCVA, as in prior studies.[11, 16, 24] *Olsen et al*. suggested that the error between US and IOLMaster measurements was significantly reduced with an SNR > 2.[16] A high degree of PSC was related with low SNR, consistent with previous reports.[11, 24] The findings in the report by *Suto et al*. revealed that severe PSC (P4 and worse) accounted for the majority of the lowest SNR group.[24]

As in our results, previous studies reported that the postoperative hyperopia could occur, especially in eyes with lower SNR.[10–14, 16, 22, 25] The preoperative AL was longer than in postoperative measurements; the difference of AL was largest in the low SNR group. Because of this measurement error, larger postoperative refraction could be predicted. *Suto et al*. suggested that severe PSC could cause scattering of light, reflected from the fundus, resulting in difficult detection.

The strength of our study is that it analyzes the presence of hyperopia in eyes with low SNR, during preoperative IOLMaster measurements. According to our results, the degree of postoperative hyperopia was approximately 0.35 D greater in group 1 at 6 months. There are 0.5 D intervals of IOL power in commercially available IOLs; therefore, the selection of 0.5 D higher power of IOL might be helpful in eyes with low SNR values. In addition, another strong point is that, in comparison with a previous study that assessed by SRK/T formula only, we used three different formulas.

In a previous study of similar subjects, the investigators divided the groups more narrowly according to SNR.[24] However, we categorized the groups for precise analysis and noted that a larger, segmented study would be helpful in more reliably assessing postoperative refraction. Notably, a previous study reported that there was no significant difference in postoperative refraction according to types of aspheric and acrylic IOLs manufactured by Abbott Medical Optics Inc. and Alcon Inc.[26] In addition, we compared three types of IOLs (Tecnis ZCB00, EnVista MX60, and AcrySof IQ SN60WF) to reduce the errors due to variety of IOLs. In Table 4, our results support that the three groups with different IOLs showed no statistical differences in MNE.

The following hypotheses can be suggested for hyperophic changes in the low SNR group. Firstly, the IOL master used in this study calculates the axial length of the cornea by using the infrared interference reflected on the front and back of the cornea.[7,9] As cataract become worse, SNR increases and lenses density increases, causing a slowdown in the speed at which light passes through the lens. However, as this is not taken into account when calculating the axial length, it is possible to overestimate axial length by having a false perception of lengthier laser travel due to prolonged travel time.

Position of the IOL can also affect the refractive power. More significant hyperopic changes were seen six months after operation than one month, which could have been affected by the posterior shifting of the IOL. Koeppl et al. found anterior movement of up to 0.3 mm in the first postoperative week. [27] There have been reports of IOL positions moving posterior by 0.033 to 0.04 mm between 1 to 6 months after cataract surgery. [28–30] In other words, the lens moved anterior short term after surgery, but from one month after surgery, a slight posterior movement occurs, which supports our conclusion of more significant hyperopic changes seen in six months after operation than one month.

There are several limitations to this study. The first is a relatively small sample size. Secondly, this study only considers MNE, and does not take into consideration postoperative axial length, anterior chamber depths, or corneal curvature. However, the most clinically important parameter in setting target refraction is the post-surgery MNE. Finally, subgroup analysis by cataract level or type was not performed. In the Low SNR group, seeing how hyperopic changes occur in eyes with severe nucleous sclerosis or eyes with PSCs can help determine the cause of the study results. But this will also require a larger sample size.

In conclusion, preoperative IOLMaster measurement of SNR between 10 and 100 could be helpful in deciding IOL power. However, if the SNR value in the preoperative measurement of IOLMaster was < 10, a hyperopic difference would be expected between postoperative spherical equivalents and target refraction anticipated before surgery. Therefore, low SNR should be considered in the selection of IOL power.
